# UCS protein function is partially restored in the *Saccharomyces cerevisiae she4* mutant with expression of the human UNC45-GC, but not UNC45-SM

**DOI:** 10.1007/s12192-017-0870-1

**Published:** 2017-12-29

**Authors:** Susana Gómez Escalante, Joseph A. Brightmore, Peter W. Piper, Stefan H. Millson

**Affiliations:** 1 Department of Molecular Biology and Biotechnology, University of Sheffield, Firth Court, Western Bank, Sheffield, S10 2TN UK; 2 School of Life Sciences, University of Lincoln, Brayford Pool, Lincoln, LN6 7DL UK

**Keywords:** UCS proteins, She4, UNC45, Hsp90, Temperature stress, Yeast

## Abstract

A dedicated UNC45, Cro1, She4 (UCS) domain-containing protein assists in the Hsp90-mediated folding of the myosin head. Only weak sequence conservation exists between the single UCS protein of simple eukaryotes (She4 in budding yeast) and the two UCS proteins of higher organisms (the general cell and striated muscle UNC45s; UNC45-GC and UNC45-SM, respectively). In vertebrates, UNC45-GC facilitates cytoskeletal functions, whereas the 55% identical UNC45-SM assists assembly of the contractile apparatus of cardiac and skeletal muscles. A *Saccharomyces cerevisiae she4Δ* mutant, totally lacking any UCS protein, was engineered to express as its sole Hsp90 either the Hsp90α or the Hsp90β isoforms of human cytosolic Hsp90. A transient induction of the human UNC45-GC, but not UNC45-SM, could rescue the defective endocytosis in these *she4Δ* cells at 39 °C, irrespective of whether they possessed Hsp90α or Hsp90β. UNC45-GC-mediated rescue of the localisation of a Myo5-green fluorescent protein (GFP) fusion to cortical patches at 39 °C was more efficient in the yeast containing Hsp90α, though this may relate to more efficient functioning of Hsp90α as compared to Hsp90β in these strains. Furthermore, inducible expression of UNC45-GC, but not UNC45-SM, could partially rescue survival at a more extreme temperature (45 °C) that normally causes *she4Δ* mutant yeast cells to lyse. The results indicate that UCS protein function has been most conserved—yeast to man—in the UNC45-GC, not UNC45-SM. This may reflect UNC45-GC being the vertebrate UCS protein that assists formation of the actomyosin complexes needed for cytokinesis, cell morphological change, and organelle trafficking—events also facilitated by the myosins in yeast.

## Introduction

The UCS (UNC45, Cro1, She4) domain-containing protein function was first identified from the study of *Caenorhabditis elegans UNC-45* (“UNCoordinated”) mutants. These display defects in motility (Barral et al. 1998; Ao and Pilgrim 2000) and cytokinesis during embryogenesis (Kachur et al. 2004). The single *C. elegans* UNC45 protein was found to associate with both Hsp90 and myosin (Barral et al. 2002); facilitating not just myosin folding but also the regulation of myosin levels by targeting excess or damaged myosin to the proteasome for degradation (Landsverk et al. 2007). It is now recognised that UCS proteins assist in the Hsp90-mediated folding of the head region of both conventional and nonconventional myosins, as well as the associations of these myosins with actin filaments.

She4, the nonessential UCS protein of budding yeast (*Saccharomyces cerevisiae*) interacts with the *S. cerevisiae* class I myosins (Myo3 and Myo5) in a temperature-dependent manner (Toi et al. 2003; Wesche et al. 2003), thereby facilitating actin cytoskeleton polarisation, endocytosis (Wendland et al. 1996) and the asymmetric mRNA localisation of *ASH1* mRNA to daughter cells (Long et al. 1997). Rng3, the corresponding UCS protein of fission yeast (*Schizosaccharomyces pombe*) interacts with both Hsp90 (Mishra et al. 2005) and the class II myosin Myo2 (Lord and Pollard 2004; Mishra et al. 2005), increasing the affinity of myosin II for actin filaments (Lord et al. 2008). *S. pombe RNG3* mutants show a 10-fold decrease in Myo5 levels, a fourfold decrease in cortical actin patches (Lord et al. 2008) and defective cytokinesis (Wong et al. 2000).

While fungi and worms have just a single UCS protein, vertebrates have two, a general cell form (UNC45-GC or Unc-45A)—expressed in most somatic cells—and a striated muscle form (UNC45-SM or Unc-45B)—highly expressed only in heart and skeletal muscle (Hutagalung et al. 2002; Price et al. 2002). These UNC45s are not functionally redundant (Comyn and Pilgrim 2012). UNC45-GC assists in the cytoskeletal functions in most cells whereas UNC45-SM has a more specific role in the assembly of the contractile apparatus of cardiac and skeletal muscles (reviewed in (Lee et al. 2014; Ni and Odunuga 2015)). Furthermore UNC45-GC interacts preferentially with the Hsp90β, not Hsp90α isoform of the human cytosolic Hsp90 (Chadli et al. 2008; Taipale et al. 2014). In contrast UNC45-SM appears to associate with both Hsp90α and Hsp90β (Etard et al. 2007; Taipale et al. 2014).

It is the characteristic C-terminal UCS domain of UCS proteins that facilitates binding of the myosin motor domain to actin filaments and the prevention of myosin aggregation (Shi and Blobel 2010). UCS proteins also possess a central domain of unknown function and—in the case of the vertebrate and worm, but not fungal UCS proteins—an N-terminal tetratricopeptide (TPR) repeat. Strong indications of how the UCS domain facilitates the association of the myosin head with its actin filament binding site have emerged from the atomic structure of the yeast She4 dimer (Shi and Blobel 2010). In addition, the *C. elegans* UNC45 has been shown to form linear multimers, a filament assembly scaffold that directly couples myosin folding with the formation of myofilaments and the organisation of sarcomeric repeats (Gazda et al. 2013).

There is only limited sequence conservation between the UCS proteins found in yeasts and higher eukaryotes (Gómez Escalante et al. 2017). We therefore investigated whether the human UNC45-GC and UNC45-SM are still able to provide UCS protein function in yeast. This report describes our analysis of whether a heterologous expression of these two human UCS proteins can rescue the defective type 1 myosin localisation, defective endocytosis and high temperature lysis defects of the *S. cerevisiae she4*Δ mutant. It also exploited *she4*Δ yeast strains lacking the native Hsp90 in which the essential Hsp90 function is provided by either the Hsp90α or Hsp90β isoform of human cytosolic Hsp90. This was to extend the investigation to a study of whether it is possible to reproduce in this simple model organism the apparent selectivity of the human UNC45-GC for Hsp90β (Chadli et al. 2008; Taipale et al. 2014).

## Materials and methods

### Yeast strains and culture

PP30*she4∆ MYO5-GFP* [pHSC82] was generated by *hphMX4* cassette (Goldstein and McCusker 1999) deletion of *SHE4* gene sequences in PP30 (MAT**a** *trp1-289 leu2-3112 his3-200 ura3-52 ade2-101*^*oc*^ *lys2-801*^*am*^ *hsc82ΔkanMX4 hsp82ΔkanMX4* [pHSC82] (Panaretou et al. 1998)); then C-terminal tagging of the *MYO5* gene with green fluorescent protein (GFP) using a *HIS3* gene cassette generated from pUG23 (Niedenthal et al. 1996). This strain has deletions of the chromosomal Hsp90 genes, but it is viable since it carries an Hsp90 gene (*HSC82*) on a *URA3* plasmid. By exchanging this *URA3* vector for a *LEU2* plasmid, three different versions of this strain—identical but for the *HSC82* promoter-directed Hsp90 gene carried on the latter *LEU2* vector—were constructed, as previously described (Millson et al. 2007). These were genes encoding either the Hsp82 isoform of the Hsp90 native to yeast (strain PP30*she4∆* [Hsp82]), the human Hsp90α (strain PP30*she4∆* [Hsp90α]) or the human Hsp90β (strain PP30*she4∆* [Hsp90β]).

### Vectors for inducible expression of 6xHis tagged She4, UNC45-GC and UNC45-SM in yeast

*UNC45-GC* and *UNC45-SM* were first amplified by PCR from cDNAs supplied as Image clones (5534906 and 40008187, respectively, from Geneservice, Cambridge, UK) using primers Hgc45F/Hgc45r and Hsm45F/Hsm45R (Table 1), respectively. In a second PCR, a 6xHis tag was added at the N-terminus of the coding sequence using primers Hgc45his6F/Hgc45r for UNC45-GC and Hsm45hisF/Hsm45R UNC45-SM. Similarly, a 6xHis tag was added at the N-terminus of the coding sequence of yeast *SHE4* gene by PCR amplification from a pRSETA clone of *SHE4* using primers HisShe4F/She4R (Table 1). All three 6xHis tagged genes were then cloned into the vector pCR®-XL-TOPO® (Invitrogen), their sequences being confirmed by terminator dye sequencing. The inserts of these pCR®-XL-TOPO-based plasmids were first inserted into the centromeric plasmid vector pShep (Gómez Escalante et al. 2017), generating vectors for their expression under the control of the promoter and terminator sequences of the yeast *SHE4* gene. Next, they were inserted into the yeast expression vector pYES2.1/V5-His-TOPO® (Invitrogen), generating vectors for their *GAL1* promoter-driven overexpression in yeast. These plasmids were inserted into the above *she4*∆ strains, selecting for growth on 2% glucose minus uracil dropout medium. Levels of expression were analysed by growing cells on 2% galactose to allow induction of protein expression, then western blot analysis with an anti-his antibody (Qiagen).

**Table 1:** PCR primers (6xHis tag-encoding sequences in italics)

Hgc45F	ATGACTGTGAGTGGTCCAGGGAC
Hgc45his6F	ATG*CATCATCATCATCATCAT*ACTGTGAGTGGTCCAGGGAC
Hgc45r	TCACTCTCCATCTTGGTTGG
Hsm45F	ATGGCAGAGGTGGAAGCGGTACA
Hsm45hisF	ATG*CATCATCATCATCATCAT*GCAGAGGTGGAAGCGGTACA
Hsm45R	CTAAGACACTGGTTTAATGAAACC
HisShe4F	ATG*CATCATCATCATCATCAT*CCACTGTGTGAGAAAGGGAA
She4R	CAAGGTACCTTAGACTTTAATTTTAGCAAGGAT

### Phenotype analysis

Conditions for the preincubation of cells with FM4-64 (a fluorescent lipophilic styryl dye that is normally rapidly internalised by endocytosis in budding yeast and then targeted to the vacuolar membrane (Smythe and Ayscough 2006)); the subsequent analysis of cells by fluorescence microscopy; also heat survival experiments were exactly as reported for a previous study (Gómez Escalante et al. 2017).

## Results

### Induction of 6xHis-She4, 6xHis-UNC45-SM and 6xHis-UNC45-GC in *she4∆* cells expressing either native or human cytosolic Hsp90

This study sought to determine whether UNC45-GC and UNC45-SM are functional in yeast; also whether it is possible to reproduce in this model organism the apparent selectivity of the human UNC45-GC for Hsp90β rather than Hsp90α (Chadli et al. 2008; Taipale et al. 2014). For this purpose we constructed yeast strains that lack the single, nonessential UCS protein of *S. cerevisiae* (She4) in which Hsp90 chaperone function is provided by one of three discrete forms of Hsp90; either a Hsp90 native to yeast (Hsp82), the human Hsp90α or the human Hsp90β (strains PP30*she4∆* [Hsp82], PP30*she4∆* [Hsp90α] and PP30*she4∆* [Hsp90β], respectively; see Methods). In addition, Myo5—one of the two class I myosins of overlapping function in *S. cerevisiae* (Myo3/5)(Goodson et al. 1996)—was GFP tagged in these *she4*Δ strains, the encoded Myo5-GFP fusion being a functional form of myosin (Gómez Escalante et al. 2017).

Initially, we inserted into strains PP30*she4∆* [Hsp90α] and PP30*she4*Δ [HSP90β] single copy genes for 6xHis-tagged forms of UNC45-GC and UNC45-SM. These had been placed under the control of the promoter and terminator sequences of the native *SHE4* gene as inserts within the single copy (centromeric) *URA3* plasmid vector pShep (Gómez Escalante et al. 2017). We also inserted into these two strains, as a control, pShep vector lacking any gene insert.

She4 is especially important for the functioning of class I myosins in yeast cells subjected to stress, since phenotypes of the *she4Δ* mutant are most readily detectable at high temperature (Goodson et al. 1996; Toi et al. 2003; Wesche et al. 2003). Functionality of the single copy 6xHis-UNC45-GC and 6xHis-UNC45-SM genes in these *she4Δ MYO5*-*GFP* transformants was therefore studied as their ability to restore Myo5-GFP localisation to cortical patches at 39 °C, as well as localisation of the fluorescent dye FM4-64 to the vacuolar membrane at this temperature, as in our earlier study of mutant forms of the native She4 in yeast (Gómez Escalante et al. 2017). However we could not obtain convincing evidence that these single copy genes were rescuing these *she4∆* phenotypes in PP30*she4∆* [Hsp90α] and PP30*she4*Δ [HSP90β] (not shown). This is despite earlier finding that the native 6xHis-*SHE4* gene on this same vector could restore She4 function in a *she4Δ* yeast mutant of a different genetic background (Gómez Escalante et al. 2017).

**Fig. 1:**
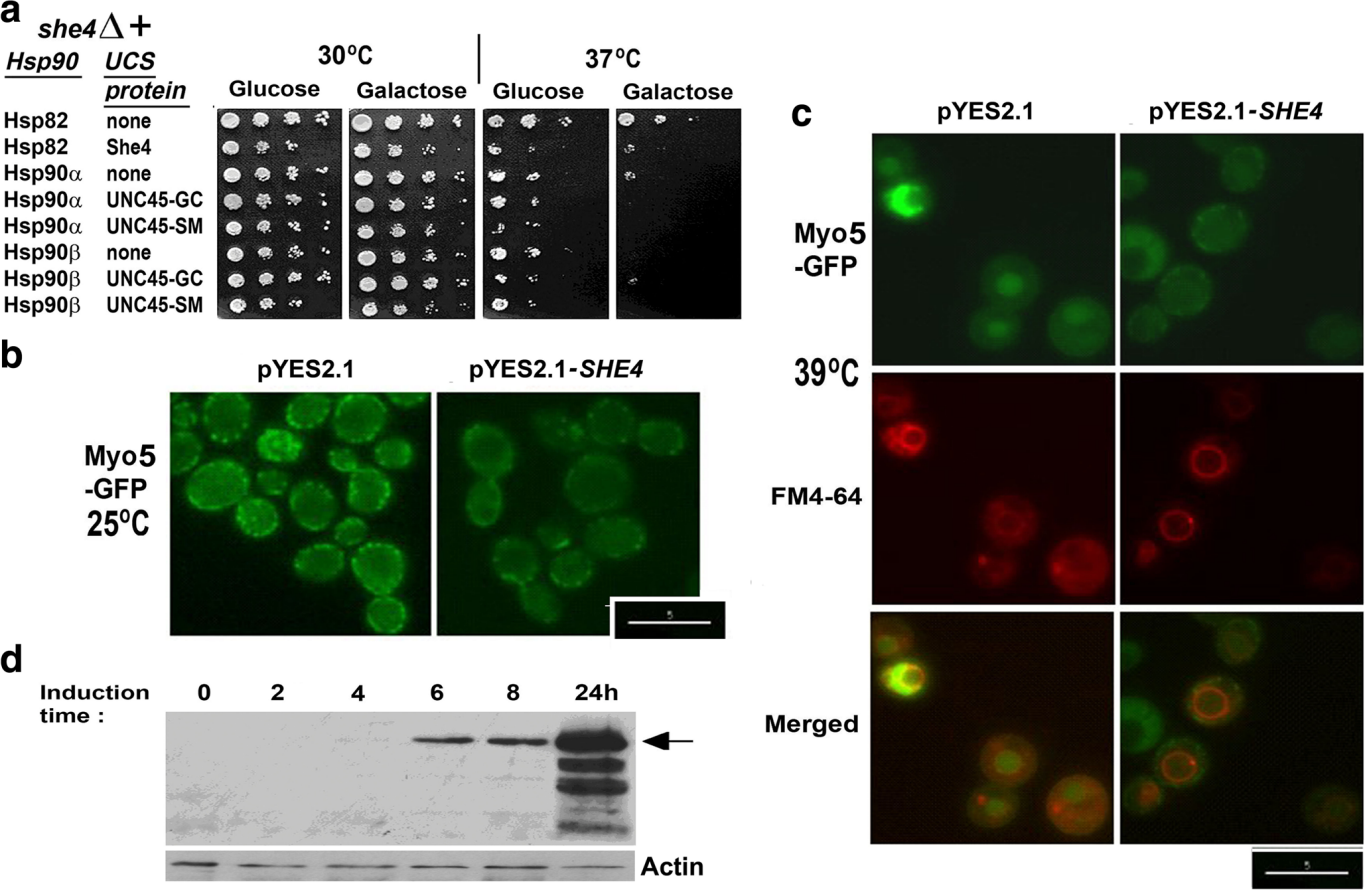
**a** Growth of PP30s*he4*Δ [HSP82], PP30*she4*Δ [HSP90α] and PP30*she4*Δ [HSP90β] bearing either the empty pYES2.1 vector (therefore expressing no UCS protein) or a pYES2.1 vector for 6xHis-She4, 6xHis-UNC45-GC and 6xHis-UNC45-SM expression. A 10-fold dilution series of 28 °C cultures in growth on minus uracil glucose medium was pinned onto minus uracil 2% glucose or galactose agar and the plates grown 3d at 30 °C and 37 °C. **b** Localisation of Myo5-GFP in PP30s*he4*Δ [HSP82] containing empty pYES2.1 and the pYES2.1 vector for 6xHis-She4 expression (pYES2.1-*SHE4*), growing on minus uracil galactose medium at 25 °C (Scale bar: 5 μm). **c** Myo5-GFP and FM4-64 localisation 1 h after the cells in (b) were heat shocked to 39 °C (scale bar 5 μm). **d** Time course of 6xHis-UNC45-GC induction in PP30 *she4*Δ [HSP90β] cells containing the pYES2.1 vector for 6xHis-UNC45-GC expression following a transfer from glucose to galactose minus uracil medium at 28 °C. 20 μg total cell protein was analysed in each gel lane, probed with anti-His and anti-actin antisera (the latter a loading control). The band corresponding to the full length 6xHis-UNC45-GC (98 kDa) is arrowed

On the assumption that these negative results might have reflected the heterologous UCS genes operating with suboptimal efficiency in yeast, we next investigated the effects of overexpressing 6xHis-UNC45-GC and 6xHis-UNC45-SM. pYES2.1-based vectors were constructed for a *GAL1-*promoter-driven overexpression of these 6xHis-tagged forms of UNC45-GC and UNC45-SM, also the native She4 protein (pYES2.1-*UNC45-GC*, pYES2.1-*UNC45-SM* and pYES2.1-*SHE4*; see Methods). Strains PP30s*he4*Δ [*HSP82*], PP30*she4∆* [Hsp90α] and PP30*she4*Δ [HSP90β] were transformed with these vectors for the overexpression of 6xHis-She4, 6xHis-UNC45-GC and 6xHis-UNC45-SM, as well as empty pYES2.1. Having confirmed that the former transformants were expressing a galactose-induced 6xHis-tagged protein of the correct size, cells were next grown on minus uracil glucose and galactose plates at 30 and 37 °C to determine whether the overexpression of 6xHis-She4, 6xHis-UNC45-GC and 6xHis-UNC45-SM would rescue the weak temperature sensitivity of these *she4*Δ transformants grown at 37 °C (Fig. 1a). Interestingly, the *GAL1* promoter-directed induction of all three proteins appeared to be slightly detrimental for growth at high temperature (Fig. 1a). In *C. elegans*, overexpression of the native UNC45 has been shown to generate defects in myosin assembly, a decreased myosin content and a mild paralysis phenotype (Landsverk et al. 2007). Similarly, overexpression of UNC45-GC in ovarian cancer cells has been correlated with increases in cell motility and metastasis (Bazzaro et al. 2007). In our hands, overexpression of the native She4 (as 6xHis-She4) in *she4*Δ cells containing the native yeast Hsp90 (strain PP30s*he4*Δ [*HSP82*]) not only failed to restore high temperature growth (Fig. 1a) but caused appreciable loss of the punctuate localisation of Myo5-GFP even at moderate temperature (Fig. 1b). At higher temperature (39 °C), it facilitated localisation of the fluorescent dye FM4-64 to the vacuolar membrane, yet caused a relatively inefficient rescue of the Myo5-GFP localisation to cortical patches (Fig. 1c and data not shown). Thus, overexpressing the native She4 in yeast generates mildly detrimental effects. These findings, together with those from other systems (Bazzaro et al. 2007; Landsverk et al. 2007), would appear to indicate that there is an optimal level for UCS protein expression in diverse cell systems.

**Fig. 2:**
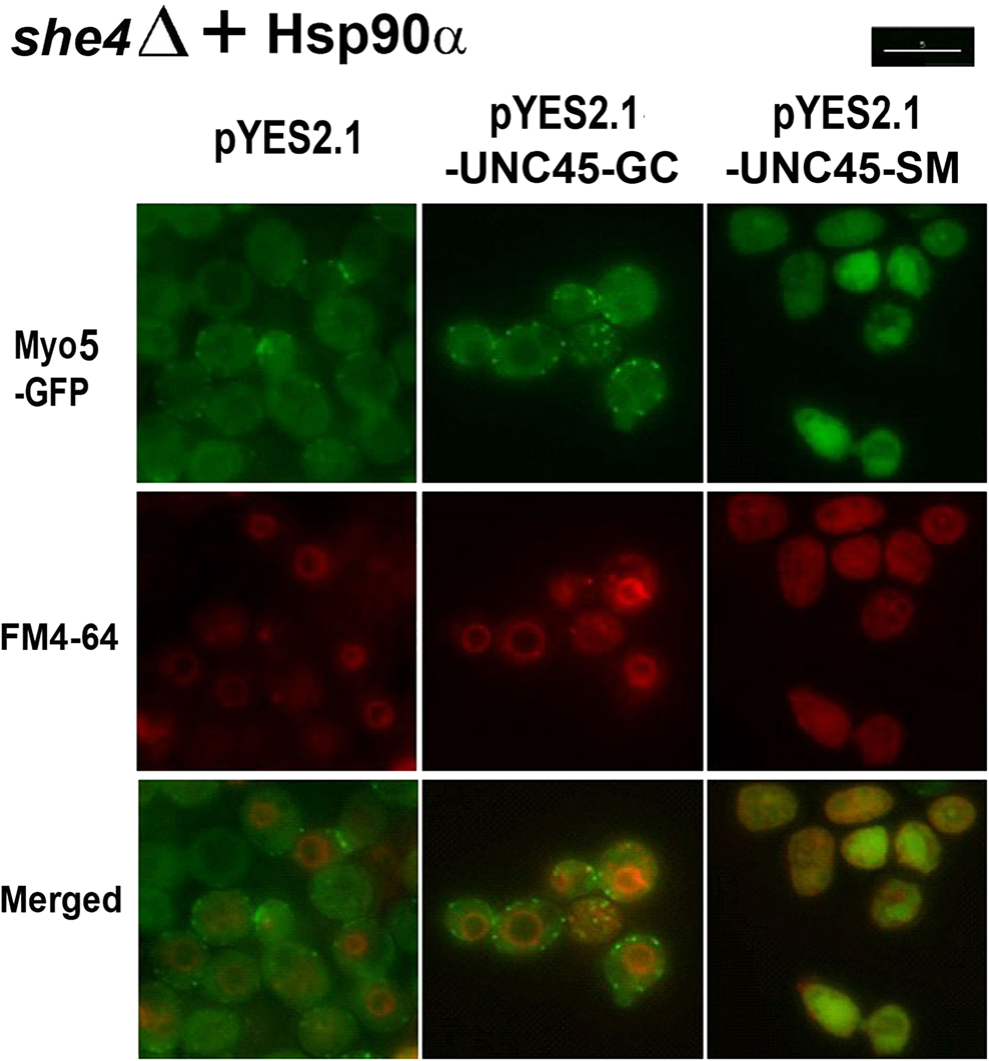
Fluorescence microscopy of Myo5-GFP and FM4-64 localisation in PP30*she4*Δ [HSP90α] containing empty pYES2.1, pYES2.1-*UNC45-GC* or pYES2.1-*UNC45-SM* 1 h after heat shock from 28 to 39 °C. Scale bar 5 μm

**Fig. 3:**
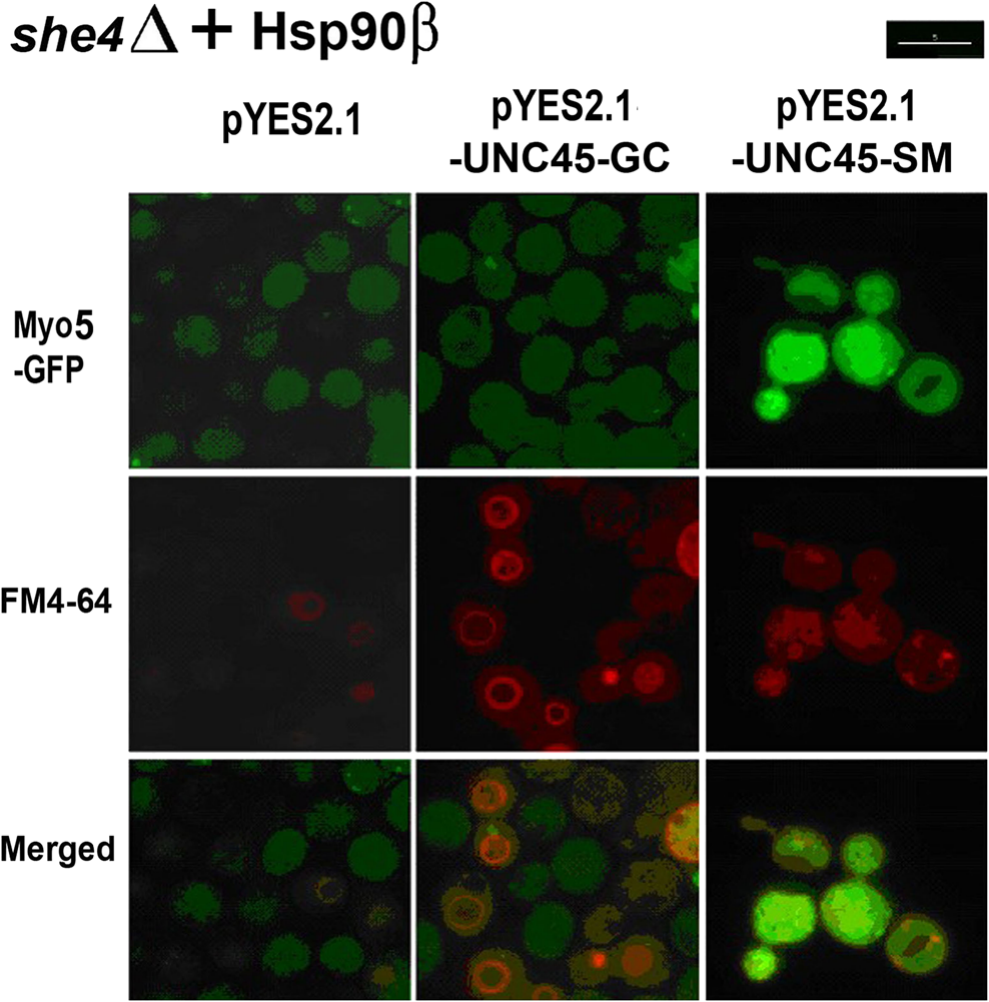
Fluorescence microscopy of Myo5-GFP and FM4-64 localisation in PP30*she4*Δ [HSP90β] containing empty pYES2.1, pYES2.1-*UNC45-GC* or pYES2.1-*UNC45-SM* 1 h after heat shock from 28 to 39 °C. Scale bar 5 μm

In view of these indications of an adverse effect of high UCS protein expression, we proceeded to analyse our pYES2.1-vector containing transformants after a transient 6xHis-UNC45-SM or 6xHis-UNC45-GC induction on galactose medium. As shown from the sample blot in Fig. 1d, protein expression from these pYES2.1-borne genes appeared optimal 6–8 h after 28 °C cultures had been transferred from glucose to galactose. Appreciable protein degradation was apparent with a 24 h induction (Fig. 1d). Therefore, it was cells induced 8 h at 28 °C on galactose minus uracil medium that were used in the subsequent microscopy of PP30*she4∆* [Hsp90α] and PP30*she4*Δ [HSP90β] transformants containing pYES2.1-*UNC45-GC* and pYES2.1-*UNC45-SM* (Figs. 2 and 3).

### Evidence for a partial rescue of *she4Δ* mutant phenotypes by 6xHis-UNC45-GC, but not 6xHis-UNC45-SM

Cultures of PP30*she4∆* [Hsp90α] and PP30*she4*Δ [HSP90β] containing either empty pYES2.1 vector, pYES2.1-*UNC45-GC* or pYES2.1-*UNC45-SM* were induced on galactose minus uracil medium for 8 h at 28 °C, then subjected to a 1 h 39 °C heat shock. As in our earlier study of mutant forms of the native She4 of yeast expressed in *she4Δ MYO5*-*GFP* cells (Gómez Escalante et al. 2017), functionality of the expressed 6xHis-UNC45-GC and 6xHis-UNC45-SM was analysed as their ability to rescue FM4-64 localisation to the vacuolar membrane and Myo5-GFP localisation to cortical patches at high temperature (39 °C). Prior to the heat shock Myo5-GFP was efficiently localised to cortical patches in all of the cultures (not shown). In the control cells containing empty pYES2.1: cells lacking any UCS protein and expressing either Hsp90α (Fig. 2) or Hsp90β (Fig. 3), this patch-like localisation of the Myo5-GFP was substantially lost at 39 °C, the Myo5-GFP becoming largely dispersed throughout the cytosol (Figs. 2 and 3). The prominent endocytosis defect in these *she4Δ* cells with empty pYES2.1 was apparent from the relatively weak FM4-64 staining of the vacuole at 39 °C, irrespective of whether such cells lacking a UCS protein expressed Hsp90α (Fig. 2) or Hsp90β (Fig. 3).

With induced 6xHis-UNC45-SM expression, we obtained no rescue of either of these phenotypes in the *she4*Δ cells with Hsp90α (Fig. 2) or Hsp90β (Fig. 3). Instead, the mislocalisations of Myo5-GFP and FM4-64 at 39 °C appeared to be rendered more enhanced by this 6xHis-UNC45-SM expression, especially in those cells that contained Hsp90α (Fig. 2). In contrast, with induced 6xHis-UNC45-GC expression there was a substantial rescue of the defective *she4Δ* cell endocytosis at 39 °C, irrespective of whether the cells contained the human Hsp90α (Fig. 2) or the human Hsp90β (Fig. 3). The corresponding rescue of Myo5-GFP localisation to cortical patches was much more prominent in the cells containing Hsp90α (Fig. 2) as compared to those that contained Hsp90β (Fig. 3). This latter effect may merely be a reflection of the apparent greater functionality of the Hsp90α isoform of human Hsp90 as compared to Hsp90β noted earlier for in cells of this yeast genetic background (Millson et al. 2007). The apparent selectivity of UNC45-GC for association with Hsp90β rather than Hsp90α in human cell extracts (Chadli et al. 2008; Taipale et al. 2014) was not reproduced in the ability of UNC45-GC to rescue *she4*Δ phenotypes this in vivo model system, since such rescue was more evident in the yeast containing—as its sole Hsp90—the human Hsp90α (Fig. 2).

### 6xHis-UNC45-GC can partially restore high temperature viability in the yeast *she4Δ* mutant

**Fig. 4:**
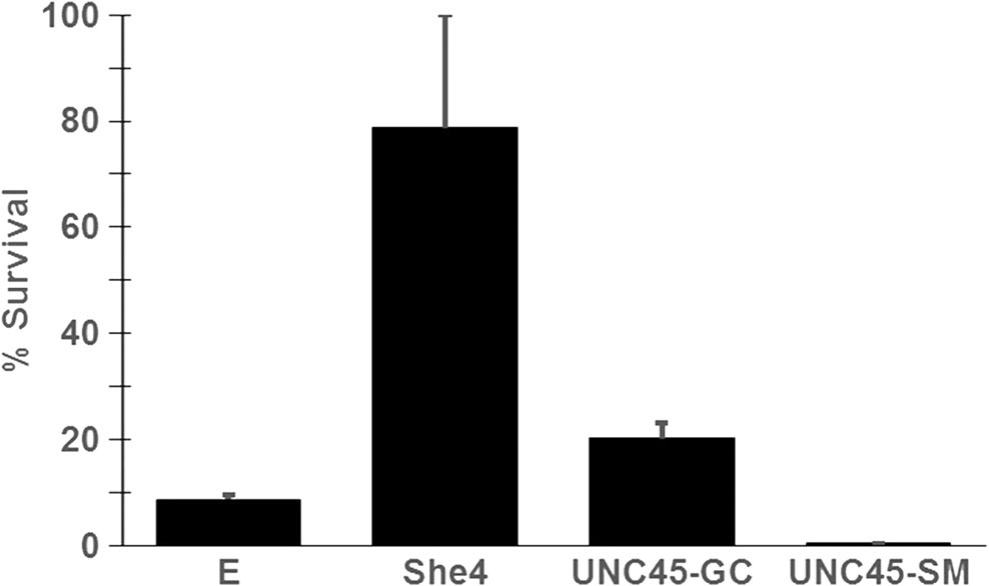
Relative survival of PP30*she4∆* [pHsp82] transformed with either empty pYES2.1 vector (E), or pYES2.1–based vectors for *GAL1-*promoter-driven expression of 6xHis-She4, 6xHis-UNC45-GC and 6xHis-UNC45-SM, induced on minus uracil galactose for 8 h at 28 °C, subjected to 1 h heat stress at 45 °C, then placed at 30 °C prior to plating in 2% glucose plates (mean and SD from six replicate experiments on each of two separate cultures of each transformant)

At the more stressful temperature of 45 °C, *she4Δ* cells undergo lysis, thereby losing viability much more rapidly than cells with a functional She4 protein (Gómez Escalante et al. 2017). We found that an induced 6xHis-UNC45-GC expression could partially restore viability to PP30*she4∆* [pHsp82] subjected to such high temperature stress, though not to the same degree as the native She4 (Fig. 4). In contrast, the corresponding expression of 6xHis-UNC45-SM appeared to be somewhat detrimental for this survival (Fig. 4). This is yet further evidence that UNC45-GC has a degree of functionality in yeast.

## Discussion

This study sought to determine whether heterologous expressions of the two human UCS proteins, UNC45-GC and UNC45-SM, can provide UCS protein function in yeast; also whether it is possible to reproduce in this model organism any apparent selectivity of UNC45-GC for Hsp90β rather than Hsp90α (Chadli et al. 2008; Taipale et al. 2014), Hsp90α and Hsp90β being the two isoforms of vertebrate cytosolic Hsp90. It exploited *she4*Δ yeast strains in which the sole, essential Hsp90 is either the native yeast Hsp82, the human Hsp90α or the human Hsp90β. Appreciable rescue of the defective endocytosis in *she4*Δ cells at 39 °C was only apparent with expression of UNC45-GC, not UNC45-SM (Figs. 2 and 3). Furthermore, it occurred irrespective of whether the yeast cells possessed—as their sole Hsp90—the human Hsp90α or Hsp90β. Induction of UNC45-GC, but not UNC45-SM, could restore type 1 myosin localisation, though this effect was more marked in the yeast containing Hsp90α rather than Hsp90β (Figs. 2 and 3). This rescue of UCS protein functions required a transient overexpression of UNC45-GC, our initial efforts to obtain rescue with a single copy UNC45-GC gene placed under the control of the promoter and terminator sequences of the native *SHE4* gene having met with no success. Such transient UNC45-GC overexpression could also restore a degree of viability to *she4*Δ cells at 45 °C (Fig. 4).

UCS proteins are dedicated cochaperones that assist in the folding and the actin association of myosins (reviewed in (Lee et al. 2014; Ni and Odunuga 2015)). Mammalian cytokinesis, cell motility, and organelle trafficking are all dependent upon the formation of actomyosin complexes that require this UNC45-GC. Since these events are also apparent in the cytoskeletal dynamics of yeast cells, it is logical to expect that protein functions (including those of She4 and UNC45-GC) needed for these processes might be more conserved in evolution, as compared to functions involved in assembly of the contractile apparatus in striated muscle tissues (such as UNC45-SM). Consistent with this, our results provide an indication that UCS protein function has been most conserved—yeast to man—in the vertebrate UNC45-GC, not UNC45-SM.

The apparent selectivity of UNC45-GC for the Hsp90β isoform of human cytosolic Hsp90, rather than Hsp90α (Chadli et al. 2008; Taipale et al. 2014) was not reproduced in this model in vivo yeast system. Probably, this is not surprising. Unlike vertebrates, yeast has just a single UCS protein, and there is no evidence for the almost identical isoforms of yeast cytosolic Hsp90 operating within separate chaperone complexes. The apparent stronger rescue of *she4*Δ phenotypes with a transient UNC45-GC overexpression in the yeast strain containing Hsp90α (Fig. 2), as compared to that with Hsp90β (Fig. 3) may merely relate to the somewhat greater functionality of the Hsp90α isoform of human Hsp90, compared to Hsp90β, as noted previously in yeast strains of this genetic background (Millson et al. 2007).
